# Residential proximity to industrial combustion facilities and risk of non-Hodgkin lymphoma: a case–control study

**DOI:** 10.1186/1476-069X-12-20

**Published:** 2013-02-22

**Authors:** Anjoeka Pronk, John R Nuckols, Anneclaire J De Roos, Matthew Airola, Joanne S Colt, James R Cerhan, Lindsay Morton, Wendy Cozen, Richard Severson, Aaron Blair, David Cleverly, Mary H Ward

**Affiliations:** 1 Division of Cancer Epidemiology and Genetics, National Cancer Institute, Department of Health and Human Services, Rockville, MD, USA; 2 TNO, Zeist, The Netherlands; 3 Department of Environmental and Radiological Health Sciences, Colorado State University, Fort Collins, CO, USA; 4 Fred Hutchinson Cancer Research Center, Seattle, WA, USA; 5 University of Washington Department of Epidemiology, Seattle, WA, USA; 6 Westat, Inc., Rockville, MD, USA; 7 Mayo Clinic College of Medicine, Rochester, MN, USA; 8 Departments of Preventive Medicine, Pathology, and Norris Comprehensive Cancer Center, Keck School of Medicine, University of Southern California, Los Angeles, CA, USA; 9 Department of Family Medicine and Karmanos Cancer Institute, Wayne State University, Detroit, MI, USA; 10 National Center for Environmental Assessment , Office of Research and Development, United States Environmental Protection Agency (retired), Washington, DC, USA; 11 Occupational and Environmental Epidemiology Branch, Division of Cancer Epidemiology and Genetics, National Cancer Institute, 6120 Executive Blvd, EPS 8006, 20892, Bethesda, MD, USA

**Keywords:** Non-Hodgkin lymphoma, Lymphomas, Dioxins, Air pollution, Geographic information systems, Case–control study

## Abstract

**Background:**

Residence near municipal solid waste incinerators, a major historical source of dioxin emissions, has been associated with increased risk of non-Hodgkin lymphoma (NHL) in European studies. The aim of our study was to evaluate residence near industrial combustion facilities and estimates of dioxin emissions in relation to NHL risk in the United States.

**Methods:**

We conducted a population-based case–control study of NHL (1998–2000) in four National Cancer Institute-Surveillance Epidemiology and End Results centers (Detroit, Iowa, Los Angeles, Seattle). Residential histories 15 years before diagnosis (similar date for controls) were linked to an Environmental Protection Agency database of dioxin-emitting facilities for 969 cases and 749 controls. We evaluated proximity (3 and 5 km) to 10 facility types that accounted for >85% of U.S. emissions and a distance-weighted average emission index (AEI [ng toxic equivalency quotient (TEQ)/year]).

**Results:**

Proximity to any dioxin-emitting facility was not associated with NHL risk (3 km OR = 1.0, 95% CI 0.8-1.3). Risk was elevated for residence near cement kilns (5 km OR = 1.7, 95% CI 0.8-3.3; 3 km OR = 3.8, 95% CI 1.1-14.0) and reduced for residence near municipal solid waste incinerators (5 km OR = 0.5, 95% CI 0.3-0.9; 3 km OR = 0.3, 95% CI 0.1-1.4). The AEI was not associated with risk of NHL overall. Risk for marginal zone lymphoma was increased for the highest versus lowest quartile (5 km OR = 2.6, 95% CI 1.0-6.8; 3 km OR = 3.0, 95% CI 1.1-8.3).

**Conclusions:**

Overall, we found no association with residential exposure to dioxins and NHL risk. However, findings for high emissions and marginal zone lymphoma and for specific facility types and all NHL provide some evidence of an association and deserve future study.

## Background

Polychlorinated dibenzo-p-dioxins (PCDDs) and polychlorinated dibenzofurans (PCDFs), commonly referred to as dioxins, are persistent organochlorine compounds generated primarily by combustion of chlorinated organic and inorganic materials [1]. PCDD/Fs are also formed inadvertently as by-products of production of chlorophenols and chlorophenoxy herbicides, bleaching of paper pulp with chlorine, and the production and use of polychlorinated biphenyls (PCBs) [1,2]. Complex mixtures of PCDD/F congeners have been found at elevated levels in soil samples near municipal waste incinerators [3-5], a major source of PCDD/F emissions to the air [6], and in house dust samples near other industrial sources of dioxins [7].

PCDDs and PCDFs have similar chemical properties and elicit their toxicological effects through a common mechanism; however, specific congeners have varying potencies [8]. The International Agency for Research on Cancer has classified the most toxic and biologically active congener, 2,3,7,8-tetrachlorodibenzo-p-dioxin (TCDD) and 2,3,4,7,8-pentachlorodibenzofuran, as human carcinogens [2,9]. The potency of a mixture of congeners is expressed in terms of the toxic equivalency quotient (TEQ), a summed metric that weights congeners relative to the potency of TCDD using toxic equivalency factors that are established for all biologically active PCDD/Fs [8] and dioxin-like PCBs.

PCDD/F exposure has been associated with an increased risk of non-Hodgkin lymphoma (NHL) in industrial cohorts [10-16] and a population in the vicinity of an accidental release [17]. Few studies have investigated NHL risk associated with environmental exposure to dioxins through air emissions. Significantly elevated NHL incidence rates were found in the vicinity of a municipal solid waste incinerator in France [18]. Two subsequent studies using Gaussian dispersion modeling to estimate air concentrations of dioxins found significant increased risks of NHL in areas with the highest predicted concentration that spanned more than 3 km [19,20]. In an ecologic study using a proximity exposure metric, NHL incidence was not significantly increased within 3 km of 72 municipal solid waste incinerators in Great Britain compared to incidence within 3 to 7.5 km [21].

The etiology of the most common NHL subtypes remains elusive, and established risk factors such as infection with the human immunodeficiency virus, specific autoimmune diseases, and high exposures to ionizing radiation explain only a small percentage of NHL occurrence [22]. The National Cancer Institute (NCI) Surveillance, Epidemiology and End Results (SEER) population-based case–control study was initiated to identify environmental risk factors for NHL and its subtypes. Within a small subset of this study population [23], we observed a significant positive association between NHL risk and blood levels of furans, dioxin-like PCBs, and the total TEQ. The aim of the current analysis was to investigate whether residential proximity to industrial sources of dioxins emissions and estimated emission levels are associated with risk of NHL.

## Methods

### NCI-SEER NHL Study

The NCI-SEER NHL Study is a population-based case–control study of NHL conducted between July 1998 and June 2000 in four U.S. SEER registry areas: the state of Iowa, Los Angeles County, and the metropolitan areas of Detroit and Seattle [24,25]. Cases were 1,321 patients with newly diagnosed and histologically confirmed NHL aged 20–74 years who did not report human immunodeficiency virus infection. Population controls (n = 1,057) were obtained by random digit dialing (under age 65) and from Medicare eligibility files (65 years and older) and were frequency matched to cases by age, sex, and race. The participation rates among eligible cases and controls were 76% and 52%, respectively; the overall response rate was 59% for cases and 44% for controls. A computer assisted personal interview that contained questions about demographic characteristics, occupational and residential history, medical conditions, and other factors was administered in the home by trained interviewers. We used a split-sample design to investigate different etiologic risk factors in detail. About half of the participants received a modified version of the Block 1995 revision of the Health Habits and History Questionnaire, a food frequency questionnaire that asked about “usual eating habits as an adult, excluding the prior year and not including any recent dietary changes”. We obtained written informed consent prior to the interview; human subjects review boards at NCI and the four study centers approved the study.

### Residential locations

As previously described [24], global positioning system (GPS) readings were taken at 95% of interview homes using a Garmin GPS12 Personal Navigator (Garmin International, Inc., Olathe, KS). Because most GPS readings were collected before the end of selective availability, GPS coordinates that were discrepant from the geocoded address by more than 200 m were checked and corrected if necessary using a combination of digital orthophotography, Census Bureau street files, road maps, and driving to the residence to collect new GPS coordinates (Seattle, Los Angeles, most of Iowa). Participants provided addresses of every home where they lived for six months or more on a residential calendar. Temporary or summer homes addresses were obtained if residence totaled two or more years. All addresses were geocoded using the TeleAtlas (Lebanon, NH) MatchMakerSDK Professional version 4.3 (October 2002) spatial database and a modified version of a Microsoft Visual Basic version 6.0 program (TeleAtlas) with a 25 foot offset from the street centerline. Addresses that were not matched were checked for errors using interactive geocoding techniques. Where only a street intersection was available for the residential location (1% of residences), we assigned the residence location to the middle of the intersection.

### Study population included in the analysis of dioxin emissions

We evaluated exposure 15 years prior to diagnosis for cases and a similar reference date for controls. This period was chosen because of the availability of data on industrial PCDD/F (hereafter dioxins) emissions for the United States in 1987 and 1995 (described below). We limited our analysis to participants with a verified GPS or geocoded residence location with a street address or intersection match for 70% or more of their person-years in the exposure period (969 cases [73%]; 749 controls [71%]).

### Dioxin emissions database

We obtained a national database of U.S. facilities and their air emissions of dioxins from the U.S. Environmental Protection Agency (EPA) (D. Cleverly, Personal communication, 2008). The database contained the facility address, latitude/longitude, and emissions (ng TEQ/year) in 1995. Facilities included secondary copper smelters, municipal solid waste incinerators, cement kilns burning hazardous waste, iron ore sintering plants, medical waste incinerators, coal-fired electric generating facilities, cement kilns burning non-hazardous waste, sewage sludge incinerators, hazardous waste incinerators, and industrial boilers. These 10 facility types accounted for over 85% of dioxin emissions from U.S. industrial sources over the past 30 years [6]. Facility locations and emissions were available in 1987 for secondary copper smelters and municipal solid waste incinerators, which had the highest dioxin air emissions in the United States.

Based on the latitude/longitude provided by EPA, 382 facilities were within 10 km of residences in our analysis. We checked the accuracy of these facility locations by comparing the coordinates to locations determined through web-based aerial photographs and ancillary information (Google Inc. Mountain View, CA; Environmental Systems Research Institute, Redlands, CA, USA); locations were corrected if necessary. We verified locations for 340 (89%) facilities and excluded 42 facilities that we could not verify. The median distance between the original and corrected location ranged from 132 meters (coal-fired electric generating facilities) to 23 km (hazardous waste incinerators).

### Estimation of emission levels over the exposure period

The database included a 1995 dioxin emission level (TEQ ng/yr) for 84% of the facilities within 5 km of our analysis population. For facilities with missing data, we assigned the average for the facility type in 1995. For facility types with only 1995 data, we assumed the facilities were operating during the entire exposure period. We estimated changes in emissions from 1983–2000 using the average emission levels for each facility type in 1987, 1995, and 2000, which we obtained from an EPA national survey of dioxin-emitting facilities [6]. We estimated the linear rate of change between 1987 and 1995, and between 1995 and 2000, by facility type. We applied the appropriate rate of change to a facility’s 1995 emission level to estimate facility-specific emission levels between 1987 and 1994 and 1996 and 2000. We assumed constant emission levels from 1983 to 1987 because air pollution controls were uncommon before 1987.

A 1987 dioxin emission level was available for all of the municipal solid waste incinerators within 5 km of our study population. When a facility was operating in 1987 but not 1995, we assumed it operated from the beginning of the exposure period to 1991 (midpoint). We assigned the 1987 level through 1991 assuming that pollution controls were not present in the final years of operation. Similarly, when a facility was operating in 1995 and not 1987, we assumed that the facility began operating in 1991. Since pollution controls were likely to have been installed during construction [6], we assigned the 1995 level from 1991 to 1995. When facilities were operating in 1987 and 1995, we assumed a linear change between 1987 and 1995, with stable emission levels before 1987 and after 1995.

In Table 1, for 1987 and 1995, we present average emission levels of all U.S. facilities, facilities within 5 and 3 km of our analysis population, the number of residences within 5 and 3 km of each facility, and the number of unique facilities. In total, 206 and 149 facilities were within 5 and 3 km, respectively, of our study population in the 15-year exposure period. No residences were within 5 km of secondary copper smelters, cement kilns burning hazardous waste, iron ore sintering plants, or industrial boilers. Emissions were highest for municipal solid waste incinerators. Medical waste incinerators and coal-fired electric generating facilities were the most common dioxin-emitting facilities and accounted for the greatest number of facility-residence pairs.

**Table 1:** **Average emission levels by facility type**^**a**^, **number of residences with within 5 and 3 km of one or more facility**, **and number of unique facilities within these distances**, **at two time points during the 15**-**year exposure period**^**b**^

	**Average emission level** (**TEQ ng**/**yr**)						**Number of facility residence pairs** (**number of unique facilities**)			
	**All U**.**S**. **facilities**		**Facilities within 5 km**		**Facilities within 3 km**		**5 km**		**3 km**	
**Facility type**	**1987**^**c**^	**1995**	**1987**^**c**^	**1995**	**1987**^**c**^	**1995**	**1987**	**1995**	**1987**	**1995**
Secondary copper smelters	327.8	327.8	-	-	-	-	0	0	0	0
Municipal solid waste incinerators	87.42	19.88	51.23	13.15	51.23	15.77	16 (2)	45 (6)	5 (2)	8 (5)
Cement kilns (hazardous waste)	6.10	8.10	-	-	-	-	0	0	0	0
Iron ore sintering plants	2.69	2.30	-	-	-	-	0	0	0	0
Medical waste incinerators	1.05	0.42	1.00	0.38	0.99	0.38	927 (120)	894 (89)	370 (95)	371 (81)
Coal-fired electric generating facilities	0.17	0.21	0.18	0.21	0.18	0.21	266 (65)	185 (38)	82 (45)	72 (31)
Cement kilns (non-hazardous waste)	0.15	0.19	0.13	0.17	0.13	0.17	47 (6)	48 (6)	16 (6)	14 (5)
Sewage sludge incinerators	0.03	0.07	0.05	0.10	0.06	0.10	66 (10)	66 (8)	23 (6)	19 (7)
Hazardous waste incinerators	0.08	0.09	0.08	0.10	0.08	0.01	19 (3)	18 (2)	2 (2)	2 (1)
Industrial boilers	0.06	0.03	-	-	-	-	0	0	0	0

^a^ Database of dioxin emitting facilities in the United States (EPA 2006).

^b^Participants with ≥70% of person-years 15 years before diagnosis/reference year with verified GPS and/or geocoded street address (includes street intersections).

^**c**^ Except for copper smelters and municipal solid waste incinerators, emission levels in 1987 were estimated from the facility’s 1995 emissions as described in the methods.

### Exposure classification

#### Proximity metrics

We evaluated the proximity of participants’ residences to dioxin-emitting facilities in the exposure period at distances of 3 and 5 km (hereafter proximity metrics). The distances were chosen based on the geographic extent of dioxin pollution plumes estimated by Gaussian models of emissions from municipal solid waste incinerators (high concentrations within 3 km and lower concentrations between 3 and 5 km) and on soil concentrations determined in other studies. Briefly, French and two Spanish studies predicted highest ground-level dioxins within about 3 km of municipal solid waste incinerators [4,5,20,26]. A US study predicted elevated concentrations within about 2 km [3]. Separately by facility type and for all facilities combined, we calculated: (a) variables for ever/never residing within 3 or 5 km of one or more facilities and (b) years of residence within these distances.

#### Emissions metrics

Mathematical models of dispersion and deposition such as the Gaussian models in the French studies [6,20] require facility-specific information such as stack height and local meteorological data. Since stack height was not available for any of the facilities in our study, we employed a simplified model that weighted the facility-specific emission by the inverse of the squared distance between the residence and the facility.

Specifically, we first calculated the inverse distance squared-weighted emission for every facility-residence pair for every year in the exposure period. For each residence, an annual emission index was then calculated by summing the inverse distance-weighted emissions over all facilities within the specified distance. If a participant lived in more than one residence in a given year, we divided the sum of the residence-specific annual emission indices for that year by the number of residences to calculate a person-specific annual emission index using the following equation:

$$\mathrm{AEI}\left(t\right)=1/x\sum_{R=1}^{x}\left[\sum_{F=1}^{n}Q_{F*}1/{\left(d_{F}\right)}^{2}\right]$$

Where,

t = specific calendar year in each study participant’s exposure period

x = the number of residences (R) for each study participant in year t

n = the number of facilities (F) within a specified distance from each residence (R) in year t

Q_F_ = the annual PCDD/F emissions (ng TEQ) for each facility

d_F_ = the distance (meters) between each residence (R) and each facility (F)

We then calculated an average AEI for each participant over their 15-year exposure period and used this as an exposure metric in our epidemiological analysis.

### Data analysis

All statistical analyses were performed in SAS Version 9.1 (SAS Institute, Cary, NC). We computed odds ratios (OR) and 95% confidence intervals (CI) using unconditional logistic regression. The reference groups were those with no facilities within the specified distance (5 or 3 km). We evaluated ever living and duration of living near dioxin emitting facilities in the 15-year exposure period for all facilities combined and separately by facility type. Duration categories for proximity metrics were zero, 1–14, and 15 years. Cut points were based on the approximate median duration of living within 3 km of any facility. We categorized the average AEI into quartiles based on the distribution among controls. All ORs were adjusted for age (<35, 35–44, 45–54, 55–64, ≥65 years), gender, race (white, black, other/unknown), study center (Detroit, Seattle, Iowa, Los Angeles), and education (<12, 12–15, ≥16 years). We also created metrics that excluded the 5 years before diagnosis for cases (reference date for controls) because recent exposures may be less likely to be associated with NHL risk. To evaluate the consistency of our findings, we evaluated the proximity and AEI metrics stratified by gender, age (<65, 65+), center and educational level (<12, 12–15, >16 years of education). We used polychotomous regression analysis to evaluate each NHL subtype separately because the etiology of NHL types may be different and previous analyses in this study population have shown varying risk factors by type [25]. ORs were calculated when there were at least 2 exposed cases.

Other potential confounders that were explored but did not materially change the ORs were family history of NHL, smoking status, body mass index, weekly alcohol consumption, and energy-adjusted monthly servings of green leafy vegetables. In addition, we evaluated potential confounding by several variables that are potential sources of dioxins including: consumption of meat, fish, and saturated fat; average population density of the participant’s census blocks over the exposure period; and duration of living within 400 meters of roads used for freight transport as a surrogate for dioxin exposure from diesel exhaust [27]. We also evaluated occupational dioxin exposure defined as working for 12 months or longer in an industry with exposure to dioxins. One or more participants had worked in petroleum refining (Standard Industrial Classification [SIC] 2911), cement/hydraulic (SIC 3241), fabricated plate work (boiler shops) (SIC 3443), electric services (SIC 4911), and refuse systems (SIC 4953). To evaluate possible selection bias, we conducted the analyses of the proximity and AEI metrics (computed for the diagnosis or reference year) among all eligible cases and controls, respectively, which included both respondents and nonrespondents. For nonrespondents, we geocoded diagnosis address for cases and mailing address for controls and limited our analysis to exact or intersection matches (927 cases, 1351 controls). For respondents, we used the home location at the time of the interview. These analyses were adjusted for age and gender only because other information was not available for nonrespondents.

## Results

Cases and controls included in our analysis were similar to the overall study population except that they had lived for a longer duration within 400 m of a freight route (Table 2). Ever working in an industry with potential dioxin exposure was associated with elevated risk of NHL in both the total NCI-SEER NHL study population (OR = 1.7, 95% CI 0.9-3.4) and our analysis population (OR = 1.8; 95% CI: 0.8-4.1).

**Table 2:** **Characteristics of cases and controls in the main NCI**-**SEER non**-**Hodgkin lymphoma study and in analysis of residential proximity to dioxin**-**emitting facilities** (**number** [**percent**], **except where indicated**)^***a***^

	**NCI-SEER NHL study**		**Study of residential proximity to dioxin-emitting facilities**^**b**^	
	**Cases (n = 1321)**	**Controls (n = 1057)**	**Cases (n = 969)**	**Controls (n = 749)**
*NHL Subtype*				
Diffuse large B-cell	417 (32)		304 (31)	
Follicular	318 (24)		227 (23)	
Chronic lymphocytic leukemia/small lymphocytic	133 (10)		102 (11)	
Marginal zone	106 (8)		82 (8)	
Other	172 (13)		128 (13)	
NOS lymphoma	175 (13)		126 (13)	
Age (years), median (IQR)	58 (48–67)	61 (50–68)	61 (50–68)	63 (53–69)
*Gender*				
Male	711 (54)	546 (52)	509 (53)	377 (50)
Female	610 (46)	511 (48)	460 (47)	372 (50)
*Race*				
Only White	1123 (85)	843 (80)	844 (87)	618 (83)
Any Black	110 (8)	151 (14)	75 (8)	99 (13)
Other/unknown	86 (7)	63 (6)	50 (5)	32 (4)
*Education level*, *years*				
<12	128 (10)	111 (11)	92 (9)	81 (11)
12-15	815 (62)	616 (58)	616 (64)	444 (59)
16+	377 (29)	330 (31)	261 (27)	224 (30)
*Study Center*				
Detroit	319 (24)	214 (20)	227 (23)	161 (22)
Iowa	361 (27)	276 (26)	282 (29)	216 (29)
Los Angeles	319 (24)	273 (26)	219 (23)	172 (23)
Seattle	322 (24)	294 (28)	241 (25)	200 (27)
*Occupational exposure to dioxins*				
Ever worked in an industry with potential dioxin exposure	27 (2.0)	13 (1.2)	20 (2.1)	9 (1.2)
*Potential sources of non-industrial exposure to dioxins*				
Average population density (people per square mile), median (IQR)	4281 (1503–7107)	4233 (1339–7572)	4261 (1369–7191)	4129 (1277–7803)
Duration of living within 400m of a freight route, years; median, IQR	0.5 (0–13)	0 (0–13)	4.5 (0–15)	4.5 (0–15)
*Sources of dietary exposure to dioxins*^c^	n = 464	n = 389	n = 316	n = 258
Meat, g/day, median (IQR)	140 (93–196)	130 (89–182)	133 (92–185)	129 (84–181)
Fish, g/day, median (IQR)	2.2 (0–6.5)	2.8 (0–6.5)	2.8 (0–6.5)	2.8 (0–6.5)
Saturated fat, mg/day, median (IQR)	24 (18–32)	22 (15–31)	23 (18–31)	23 (15–32)

IQR interquartile range.

^a^Percentages do not add to 100 due to missing data; 1 case in the main study was missing education information.

^b^Participants with ≥70% of person-years 15 years before diagnosis/reference year with verified GPS and/or geocoded street address (including street intersections).

^c^Dietary questionnaires were available for a subset of the population as indicated in the table.

Thirty-nine percent of cases and controls had lived within 5 km of one or more dioxin-emitting facility during the 15-year period (Table 3). Percentages for 3 km were 22% and 23% for cases and controls, respectively. Medical waste incinerators were the most common facility within 5 km (32% of cases, 33% of controls). The percentage of cases and controls residing near other facility types was substantially lower. We found no association between ever residing within 5 or 3 km of any facility and NHL risk, and no association with duration. We observed an elevated risk for residence <5 km from cement kilns (OR = 1.7, 95% CI 0.8-3.3) that increased for residence <3 km (OR = 3.8, 95% CI: 1.1-14.0); the association was stronger for a residence period of 1–14 years than for 15 years. Residence within 5 km of a hazardous waste incinerator was associated with a nonsignificantly elevated NHL risk (OR = 1.4, 95% 0.6-3.6). Living within 5 km of a municipal solid waste incinerator was associated with significantly reduced NHL risk (OR = 0.5, 95% CI = 0.3-0.9), particularly at 1–14 years duration (OR = 0.4, 95% CI = 0.2-0.8). Only two and three cases lived <3 km from hazardous waste and solid waste incinerators, respectively.

**Table 3:** Residential proximity to dioxin emitting facilities and risk of NHL

	**Ever lived within 5 km **^**a**^			**Years lived within 5 km**				**Ever lived within 3 km**^**a**^			**Years lived within 3 km**			
**Facility type**	**Cases N (%)**	**Controls N (%)**	**OR (95% CI)**^**b**^	**Years**	**Cases**	**Controls**	**OR (95% CI )**^**b**^	**Cases N (%)**	**Controls N (%)**	**OR (95% CI)**^**b**^	**Years**	**Cases**	**Controls**	**OR (95% CI)**^**b**^
Any dioxin- emitting facility	374	290	1.0 (0.8-1.2)	0	595	459	1.0	215	170	1.0 (0.8-1.3)	0	754	579	1.0
	(39)	(39)		1-14	158	110	1.0 (0.8-1.4)	(22)	(23)		1-14	112	90	0.9 (0.7- 1.3)
				15	216	180	0.9 (0.7-1.2)				15	103	80	1.1 ( 0.8-1.5)
Medical waste incinerators	312	250	0.9 (0.7-1.2)	0	657	499	1.0	185	154	0.9 (0.7-1.2)	0	784	595	1.0
	(39)	(33)		1-14	125	96	0.9 (0.7-1.3)	(19)	(21)		1-14	93	82	0.8 (0.6-1.2)
				15	187	154	1.0 (0.7-1.3)				15	92	72	1.1 (0.8-1.5)
Coal-fired electric generating facilities	56	45	0.9 (0.6-1.3)	0	913	704	1.0	25	23	0.7 (0.4-1.3)	0	944	726	1.0
	(5.8)	(6.0)		1-14	24	23	0.7 (0.4-1.2)	(2.6)	(3.1)		1-14	13	15	0.6 (0.3-1.2)
				15	32	22	1.1 (0.6-1.9)				15	12	8	1.1 ( 0.4- 2.8)
Sewage sludge incinerators	52	33	1.1 (0.7-1.7)	0	917	716	1.0	19	12	1.1 (0.5-2.3)	0	950	737	1.0
	(5.4)	(4.4)		1-14	28	15	1.2 (0.6-2.3)	(2.0)	(1.6)		1-14	13	4	2.2 (0.7-7.1)
				15	24	18	1.0 (0.5-1.8)				15	6	8	0.5 (0.2-1.5)
Municipal solid waste incinerators	18	28	0.5 (0.3-0.9)	0	951	721	1.0	3	7	0.3 (0.1-1.4)	0	966	742	1.0
	(1.9)	(3.7)		1-14	14	24	0.4 (0.2-0.8)	(0.3)	(0.9)		1-14	2	5	0.3 (0.06-1.8)
				15	4	4	0.9 (0.2-3.5)				15	1	2	--
Cement kilns (non-hazardous)	27	13	1.7 (0.8-3.3)	0	942	736	1.0	13	3	3.8 (1.1-14.0)	0	956	746	1.0
	(2.8)	(1.7)		1-14	14	4	2.6 (0.8-8.1)	(1.3)	(0.4)		1-14	6	0	>999
				15	13	9	1.2 (0.5-3.0)				15	7	3	2.1 (0.5-8.7)
Hazardous waste incinerators	14	7	1.4 (0.6-3.6)	0	955	742	1.0	2	2	0.7 (0.1-5.1)	0	967	747	1.0
	(1.4)	(0.9)		1-14	6	3	1.4 (0.3-5.8)	(0.2)	(0.3)		1-14	2	2	0.7 (0.1-5.1)
				15	8	4	1.4 (0.4-4.8)				15	0	0	--

^a^ Reference groups: did not live within specified distance in the exposure period.

^b^Adjusted for age (<35, 35–44, 45–54, 55–64, >65 yrs), gender, race (white, black, other/unknown), center (Detroit, Seattle, Iowa, Los Angeles), education (<12, 12–15, >16 years).

We observed no association between quartiles of the AEI and risk of NHL overall (Table 4). Risk of marginal zone lymphoma was increased for the highest AEI quartile at 5 km (OR = 2.6, 95% CI 1.0-6.8) and 3 km (OR = 3.0, 95% CI 1.1-8.3) but not for lower exposure quartiles. The AEI was not associated with other NHL subtypes. We observed no significant associations between residence within 5 and 3 km of any facility and any NHL subtype. Power was limited to evaluate residential proximity to specific facilities by subtype.

**Table 4:** **Proximity to any facility**, **the average annual dioxin emission index**, **and risk of non**-**Hodgkin Lymphoma**: **all NHL and by histological subtype**

	**Never lived within distance**			**Ever lived within distance**			**Average annual emission index**											
							**Quartile 1**			**Quartile 2**			**Quartile 3**			**Quartile 4**		
	**Cases**	**Controls**	**OR (95%CI)**^**a**^	**Cases**	**Controls**	**OR (95%CI)**^**a**^	**Cases**	**Controls**	**OR (95%CI)**^**a**^	**Cases**	**Controls**	**OR (95%CI)**^**a**^	**Cases**	**Controls**	**OR (95%CI)**^**a**^	**Cases**	**Controls**	**OR (95%CI)**^**a**^
**5 km**																		
All NHL	595	459	1.0	374	290	1.0 (0.8-1.2)	97	73	1.0 (0.7-1.4)	102	72	1.1 (0.7-1.5)	85	73	0.9 (0.6-1.3)	90	72	1.0 (0.7-1.4)
DLBCL	182	459	1.0	122	290	1.0 (0.7-1.3)	30	73	1.0 (0.6-1.6)	28	72	0.9 (0.5-1.5)	32	73	1.0 (0.6-1.6)	32	72	1.0 (0.6-1.7)
Follicular	141	459	1.0	86	290	1.0 (0.7-1.4)	22	73	0.9 (0.5-1.6)	26	72	1.1 (0.7-1.9)	18	73	0.8 (0.4-1.5)	20	72	1.0 (0.5-1.7)
CLL/SLL	66	459	1.0	36	290	0.8 (0.5-1.3)	9	73	0.9 (0.4-2.0)	8	72	0.7 (0.3-1.6)	9	73	0.8 (0.3-1.7)	10	72	0.9 (0.4-1.9)
Marginal zone	57	459	1.0	25	290	1.1 (0.6-1.8)	5	73	0.5 (0.2-1.5)	10	72	1.5 (0.7-3.3)	2	73	0.5 (0.1-2.2)	8	72	2.6 (1.0-6.8)
Other subtypes	78	459	1.0	50	290	0.8 (0.5-1.3)	15	73	1.2 (0.6-2.3)	12	72	0.8 (0.4-1.6)	13	73	0.7 (0.3-1.5)	10	72	0.5 (0.2-1.2)
NOS lymphoma	71	459	1.0	55	290	1.4 (0.9-2.2)	16	73	1.4 (0.8-2.7)	18	72	1.8 (1.0-3.3)	11	73	1.1 (0.5-2.4)	10	72	1.1 (0.5-2.5)
**3 km**																		
All NHL	754	579	1.0	215	170	1.0 (0.8-1.3)	67	43	1.2 (0.8-1.8)	39	42	0.7 (0.5-1.2)	52	43	0.9 (0.6-1.4)	57	42	1.1 (0.7-1.7)
DLBCL	235	579	1.0	69	170	0.9 (0.6-1.3)	22	43	1.2 (0.7-2.1)	11	42	0.6 (0.3-1.2)	14	43	0.7 (0.4-1.3)	22	42	1.2 (0.7-2.1)
Follicular	178	579	1.0	49	170	1.0 (0.6-1.4)	18	43	1.3 (0.7-2.5)	6	42	0.5 (0.2-1.1)	11	43	0.8 (0.4-1.7)	14	42	1.1 (0.6-2.2)
CLL/SLL	81	579	1.0	21	170	0.9 (0.5-1.6)	3	43	0.6 (0.2-1.9)	4	42	0.7 (0.2-2.0)	9	43	1.7 (0.7-3.7)	5	42	0.8 (0.3-2.3)
Marginal zone	66	579	1.0	16	170	1.3 (0.7-2.4)	3	43	0.7 (0.2-2.3)	4	42	1.3 (0.4-3.9)	3	43	1.1 (0.3-4.0)	6	42	3.0 (1.1-8.3)
Other subtypes	101	579	1.0	27	170	0.8 (0.5-1.3)	8	43	1.0 (0.5-2.3)	8	42	1.0 (0.4-2.2)	6	43	0.6 (0.3-1.6)	5	42	0.6 (0.3-2.5)
NOS lymphoma	93	579	1.0	33	170	1.5 (0.9-2.3)	13	43	2.1 (1.1-4.2)	6	42	1.1 (0.4-2.7)	9	43	1.5 (0.6-3.2)	5	42	0.9 (0.3-2.5)

DLBCL = Diffuse large B-cell lymphoma; CLL/SLL = Chronic lymphocytic leukemia/small lymphocytic lymphoma; NOS lymphoma = not otherwise specified.

^a^ Adjusted for age (<35, 35–44, 45–54, 55–64, >65 yrs), gender, race (white, black, other/unknown), center (Detroit, Seattle, Iowa, Los Angeles), years of education (<12, 12–15, >16).

Lagging the exposure period by 5 years produced results similar to unlagged analyses. There were no notable differences in the associations for the proximity metrics and the AEI across categories of gender, age, and education (not shown). Distributions of the exposure metrics varied by center. Among controls, 74% of Detroit participants lived within 5 km of a facility and 32% were in the highest AEI quartile. These percentages were 44% and 0.5%, respectively, for Seattle, 31% and 9% for Iowa, and 9% and 0% for Los Angeles. Some of the ORs for the proximity metrics varied by center. ORs for <5 km from cement kilns were OR = 2.1 (95% CI 0.4-11.3) for Detroit, OR = 1.9 (95% CI 0.8-4.7) for Seattle, and OR = 0.7 (95% CI 0.1-3.4) for Iowa. Los Angeles had no participants living <5 km from cement kilns. The association with proximity to hazardous waste incinerators was specific to Los Angeles (OR = 1.7, 95% CI 0.6-4.5), as only one control in Seattle lived near these facilities and no cases and controls did in Iowa or Detroit. The inverse association with residence near municipal solid waste incinerators was observed for all centers except Seattle (2 cases, 1 control).

A lower proportion of nonrespondents lived within 5 km of a dioxin-emitting facility (26% for both cases and controls) than our analysis population (39% for both cases and controls at diagnosis and reference date, respectively). However, based on the current residence, the association between proximity to any facility and NHL risk did not differ by participation status. Associations for specific facility types were similar except that we observed no association with proximity to municipal solid waste incinerators (5 km OR = 0.9, 95% CI 0.6-1.2; 3 km OR = 0.8, 95% CI 0.4-1.5) among all eligible cases and controls. Among this group, a weaker association was observed for residence within 5 and 3 km of cement kilns (5 km OR = 1.1, 95% CI 0.7-1.7; 3 km OR = 1.9, 95% CI 0.9-4.1). Residence within 5 km of hazardous waste incinerators (OR = 2.2, 95% CI 1.2-3.8) but not 3 km (OR = 1.2, 95% CI 0.4-3.4) was associated with increased risk of NHL. Among all eligible cases and controls, risks for marginal zone lymphoma associated with the highest quartile of the AEI were attenuated (5 km OR = 0.9, 95% 0.5-1.6; 3 km OR = 1.3, 95% CI 0.6-2.7).

## Discussion

We observed no association between residence within 5 or 3 km of one or more dioxin-emitting facilities and NHL risk. However, we observed significantly elevated risk of NHL for individuals living within 3 km of cement kilns and an inverse association with proximity to municipal solid waste incinerators. The positive association for cement kilns did not appear to be due to selection bias based on our analyses of current residence for all eligible cases and controls; whereas, we found no association with proximity to municipal solid waste incinerators among this group. Our emission metric, which incorporated facility-specific dioxin emissions within 3 or 5 km of residences, was not associated with NHL risk overall or most NHL subtypes. Risk of marginal zone lymphoma was increased among those in the highest quartile, but there was no trend and the association was attenuated in analyses of all eligible cases and controls.

Our results do not support earlier observations that living near municipal solid waste incinerators is associated with increased risk of NHL [18]; however, our power to evaluate this association was limited due to the small numbers of study participants living within 5 km of these facilities. In a French study, NHL risk was significantly increased (OR = 2.3, 95% CI 1.4-3.8) for residence in the highest exposure zone (an area approximately 1 km by 4 km based on a Gaussian model) around a facility with high emissions (16.3 ng I-TEQ/m^3^ in 1998) that exceeded the European Union Standard of 0.1 ng I-TEQ/m^3^[19]. The results from a subsequent case–control study [28] that estimated serum levels of the 1998 World Health Organization-TEQ among residents near this incinerator further support an association between environmental exposure to dioxins from municipal solid waste incinerators and risk of NHL. An ecological study of NHL risk and residence near 13 incinerators with lower emissions in France reported increased risk only among women (RR = 1.18, 95% CI 1.01-1.37) [20]. Our emission metric was an index based on mass released per year (ng TEQ/yr) rather than concentration at a distance (ng TEQ/m^3^), which was the basis for French estimates. Therefore, it was not possible to directly compare exposure levels. A small area ecological study in Great Britain found no association between NHL incidence and distance from incinerators (within 3 km of 72 municipal solid waste incinerators compared to 3 to 7.5 km) [21]. Differing results across studies may be due to different study designs, emission levels, and exposure misclassification.

We found no published studies that evaluated risk of NHL associated with environmental exposure to cement kiln emissions. The U.S. EPA average emission level for this facility type was lower than solid waste incinerators and some other facility types (Table 1). However, the TEQ is heavily weighted by PCDDs and 2,3,7,8-tetrachlorofuran dominates emissions from cement kilns (non-hazardous) [29]. We conducted a pilot study to evaluate determinants of dioxins and furans in house dust in a subset of our study homes [30]. Four facility types were located near the pilot study homes: non-hazardous waste cement kilns, coal-fired power plants, sewage sludge incinerators, and medical waste incinerators. We found 2- to 9-fold higher concentrations of 5 PCDDs and 5 PCDFs in homes within 5 km of these cement kilns compared with homes further away, which provides some evidence that our findings may be related to these exposures. With the exception of higher TCDD levels in homes within 5 km of sewage sludge incinerators, proximity to the other three facility types was not association with PCDD and PCDF concentrations in homes.

Dietary exposure to dioxins, mainly through consumption of meat, milk, eggs and fish, is generally believed to be the most important source of non-occupational dioxin exposure [2]. Dioxin levels in blood and breast milk in non-occupationally exposed subjects residing near incinerators in Spain and Germany were not elevated in those who lived in close proximity to incinerators compared with those living farther away [31-34]. However, a recent Russian study found higher dioxin levels among women living within a few kilometers of a dioxin-emitting chemical plant and eating locally-produced foods [35]. Similarly, in a French study, there was no association between serum dioxin levels and residential proximity to a municipal waste incinerator except among those who consumed animal products produced locally [36]. Exposure routes were not determined in the previous positive studies [18-20], but it is possible that NHL associations with residential proximity were driven by local food consumption. Although dietary information was collected in our study, no information on local food consumption was available.

Our study had several strengths. We evaluated NHL risk in relation to a nationwide database of dioxin-emitting facilities, which allowed us to investigate several industrial sources separately and account for exposure from multiple sources simultaneously. Our residential histories allowed us to account for the mobility of the study population and to include information on changes in emissions over time. Previous studies were registry-based and were limited to the residence at diagnosis. Additionally, we had individual level information on potential NHL risk factors, while previous studies had limited data on potential confounders.

Our study had several limitations including small numbers for analyses of specific facility types and NHL subtypes. With the exception of municipal solid waste incinerators, facility-specific emissions were available only for 1995. We estimated changes in the facility’s emissions resulting from regulations in the late 1980s and early 1990s using an EPA national survey of facility-specific changes in air emissions [6]. However, we did not have information on the years in which the facilities started and ceased to be operational, which may have led to misclassification of exposure. We estimated exposure 15 years before diagnosis; however, the latency of NHL in relation to environmental exposures is not known and earlier exposures may be important. Another limitation that may have resulted in exposure misclassification was the relatively simple proximity and distance-weighted emission metrics, which may not capture the complex and asymmetric exposure patterns that can result from local meteorological, facility, and terrain characteristics. Two of the prior studies included stack height, meteorological data, and surface topography, enabling exposure modeling using a Gaussian-type dispersion model [19,20]; however, we did not have stack height for the facilities in our study. The amount of resulting exposure misclassification in our study depends on how much these factors influenced the true spatial distribution of dioxin emissions.

Our response rates were low especially among controls. However, analyses based on the current residence of all eligible cases and controls provided support for most of the associations we observed. Further, we found no notable differences in the associations across categories of gender, age, and education, factors associated with nonresponse in this population [37]. Finally, the positive findings we observed should be interpreted with caution because we made many comparisons and some associations were based on small numbers.

## Conclusions

In summary, our findings do not provide substantial evidence that residential exposure to industrial dioxin emissions in these areas of the United States increases NHL risk. We were able to evaluate multiple sources of exposure and to account for residential mobility, improvements over previous studies. In spite of the overall null findings, the positive associations we observed deserve further study. NHL risk was positively associated with residence near cement kilns. Risk of marginal zone lymphoma was significantly increased among those with the highest residential exposure to dioxin emissions as estimated across all facility types. Future research should overcome the limitations of this study by incorporating a metric for local food consumption, focusing on estimating risk across a gradient of exposure including facilities with the highest emissions, and modeling air emissions using meterological data and facility characteristics such as stack height.

## Abbreviations

CI: Confidence interval; EPA: Environmental Protection Agency; GPS: Global positioning system; NCI: National Cancer Institute; NHL: Non-Hodgkin lymphoma; OR: Odds ratios; PCBs: Polychlorinated biphenyls; PCDDs: Polychlorinated dibenzo-p-dioxins; PCDFs: Polychlorinated dibenzofurans; TCDD: 2,3,7,8-tetrachlorodibenzo-p-dioxin; SEER: Surveillance, Epidemiology and End Results; SIC: Standard Industrial Classification; TEQ: Toxic equivalency quotient; US: United States

## Competing interests

The authors declare that they have no competing interests.

## Authors’ contributions

AP participated in the exposure assessment, carried out the primary data analysis, and prepared the first draft of the manuscript. JRN led the exposure assessment and participated in writing the manuscript. AJD helped to coordinate the data collection and participated in the manuscript preparation. MA conducted the GIS analysis and participated in the manuscript preparation. JSC, JRC, RS, and WC participated in the original study design, coordination of data collection and in the manuscript preparation. LM coordinated the original study and participated in the manuscript preparation. AB participated in the study design and manuscript preparation. DC provided the EPA dioxin emissions database and provided advice about the exposure assessment. MHW participated in the original study design, data collection, coordination of this analysis, and prepared later drafts of the manuscript. All authors read and approved the final manuscript.
