# Correction to: Residential proximity to industrial combustion facilities and risk of non-Hodgkin lymphoma: a case-control study

**DOI:** 10.1186/s12940-021-00798-9

**Published:** 2021-11-01

**Authors:** Anjoeka Pronk, John R. Nuckols, Anneclaire J. De Roos, Matthew Airola, Joanne S. Colt, James R. Cerhan, Lindsay Morton, Wendy Cozen, Richard Severson, Aaron Blair, David Cleverly, Mary H. Ward

**Affiliations:** 1 Division of Cancer Epidemiology and Genetics, National Cancer Institute, Department of Health and Human Services, Rockville, MD USA; 2 TNO, Zeist, The Netherlands; 3 Department of Environmental and Radiological Health Sciences, Colorado State University, Fort Collins, CO USA; 4 Fred Hutchinson Cancer Research Center, Seattle, WA USA; 5 University of Washington Department of Epidemiology, Seattle, WA USA; 6 Westat, Inc., Rockville, MD USA; 7 Mayo Clinic College of Medicine, Rochester, MN USA; 8 Departments of Preventive Medicine, Pathology, and Norris Comprehensive Cancer Center, Keck School of Medicine, University of Southern California, Los Angeles, CA USA; 9 Department of Family Medicine and Karmanos Cancer Institute, Wayne State University, Detroit, MI USA; 10 National Center for Environmental Assessment, Office of Research and Development, United States Environmental Protection Agency (retired), Washington, DC, USA; 11 Occupational and Environmental Epidemiology Branch, Division of Cancer Epidemiology and Genetics, National Cancer Institute, 6120 Executive Blvd, EPS 8006, Bethesda, MD 20892 USA

**Correction to: Environ Health 12, 20 (2013)**

**https://doi.org/10.1186/1476-069X-12-20**

Following publication of the original article [1], the authors reported an error in the unit of the average emission metric (AEI). The AEI was incorrectly reported as nanograms (ng) toxic equivalency quotient (TEQ)/year in the abstract, methods, discussion, and Table 1. The correct unit is grams (g) TEQ/year. The authors apologize for this error.
